# Prognostic Impact of Baseline Six-Minute Walk Distance following Trans-Catheter Aortic Valve Replacement

**DOI:** 10.3390/jcm12072504

**Published:** 2023-03-26

**Authors:** Teruhiko Imamura, Nikhil Narang, Ryuichi Ushijima, Mitsuo Sobajima, Nobuyuki Fukuda, Hiroshi Ueno, Koichiro Kinugawa

**Affiliations:** 1 The Second Department of Internal Medicine, University of Toyama, 2630 Sugitani, Toyama 930-0194, Japan; 2 Advocate Christ Medical Center, Oak Lawn, IL 60453, USA

**Keywords:** heart failure, hemodynamics, exercise, aortic valve disease

## Abstract

Background: The six-minute walk test is a practical tool for assessing functional capacity in patients with a variety of etiologies including pulmonary disease and heart failure. Six-minute walk distance (6MWD) is associated with mortality and morbidity in patients with a variety of comorbidities. We aimed to assess the prognostic impact of baseline 6MWD in patients with severe aortic stenosis undergoing trans-catheter aortic valve replacement (TAVR). Methods: Patients with severe aortic stenosis who underwent a six-minute walk test after index admission and underwent TAVR between 2015 and 2022 were included in this retrospective study. Patients were followed up for two years or until November 2022 following TAVR. The impact of baseline 6MWD on the primary composite outcome, defined as all-cause death and all-cause readmission during the 2-year observation period following index discharge, was assessed. Results: A total of 299 patients (median age 86 years old, 85 men) were included. They received a 6-min walk test prior to TAVR, underwent successful TAVR procedures, and were discharged alive. The median baseline 6MWD was 204 (143, 282) meters. Shorter baseline 6MWD was associated with higher cumulative incidence of the primary outcome with an adjusted hazard ratio of 0.76 (95% confidence interval 0.58–1.01, *p* = 0.055) with a cutoff 6MWD of 178 m during the 2-year observation period after index discharge. Conclusions: Overall, functional capacity was impaired in the elderly patients with severe aortic stenosis prior to TAVR. Patients with severe aortic stenosis having shorter baseline 6MWD tended to have higher rates of morbidity and mortality after successful TAVR. The clinical implication of aggressive cardiac rehabilitation to improve patients’ functional capacity and 6MWD-guided optimal patient selection remain the future concerns.

## 1. Background

Trans-catheter aortic valve replacement (TAVR) is now the gold standard therapy for severe aortic stenosis in high-risk elderly patients with proven excellent clinical outcomes at high-volume centers [1]. In addition, clinical trials are exploring the utility of TAVR as a non-inferior option to surgical aortic valve replacement in intermediate- and low-risk surgical cohorts with severe aortic stenosis [2]. Nevertheless, steps are needed to reduce mortality and morbidity after TAVR, and a primary way to better achieve this is to better understand optimal patient selection [3].

Baseline comorbidities such as chronic kidney disease and physical frailty are strong predictors of outcomes following TAVR [4,5]. Frailty can manifest as nutritional deficiencies or functional limitations. However, there is debate as to which criteria best define frailty. Several available clinical frailty scales are limited by complexity in calculation, making them challenging for routine clinical use in most community settings [6].

Clinical outcomes are closely related to functional capacity in heart failure patients. Peak oxygen consumption at cardiopulmonary exercise test is the gold standard for the evaluation of functional capacity. However, cardiopulmonary exercise tests are rarely applied to most heart failure patients, due to limited facilities for the tests and their multiple comorbidities.

Therefore, the 6-min walk test (6MWT) has been proposed as a simple, inexpensive, and reproducible alternative to cardiopulmonary exercise testing. The 6MWT is a standardized field test used to assess functional exercise capacity in patients with a variety of chronic diseases, including chronic heart failure [7]. The 6MWT can replicate activities of daily life and is particularly relevant for the elderly patients, who typically present with symptoms below their theoretical maximal exercise capacity. The advantages of 6MWT are its ease of use and established benchmarks associated with clinical risk.

Taken together, the 6MWT could theoretically be a promising, reproducible, and simple tool to estimate functional capacity and risk-stratify the elderly patients with severe aortic stenosis undergoing TAVR. Hence, we evaluated the use of 6MWT as an indicator of frailty and its associated prognostic impact in patients with severe aortic stenosis undergoing TAVR.

## 2. Methods

### 2.1. Patient Selection

Consecutive patients with severe aortic stenosis who were admitted to our institution for TAVR between 2015 and 2022 were enrolled in our prospective registry database and considered for their eligibility for this study. Patients generally received 6MWT on admission according to our institutional protocol. Detailed inclusion and exclusion criteria are summarized in Table 1. Of these, patients who were unable to tolerate 6MWT due to a preexisting comorbid condition, including stroke, peripheral arterial disease, and chronic obstructive pulmonary disease, did not receive 6MWT and were excluded from the analysis. Patients receiving continuous intravenous inotropes were excluded because of their potential impact on exercise capacity. Patients who died during the index hospitalization were also excluded, as there was no observation period after the index discharge. Written informed consent was obtained from all participants at enrollment for inclusion in our institutional database and for use of their data in clinical studies. The institutional review board approved the study protocol.

### 2.2. 6MWT

On admission, the 6MWT was carried out in a closed corridor in a standard manner by experienced cardiologists who were blinded to the study protocol [8]. Two markers were placed on the floor at 30 m intervals and patients walked from one end to the other for 6 min. Patients were instructed to walk as fast as possible and were informed of the time elapsed on each lap. The total distance that patients walked for six minutes was recorded as a six-minute walk distance (6MWD).

### 2.3. TAVR Procedure

Patients with severe aortic stenosis with peak velocity >4.0 m/s, mean pressure gradient >40 mmHg, or aortic valve area <1.0 cm^2^ were considered for TAVR after the multidisciplinary heart-valve team conference. All patients in this study met the indication for TAVR and agreed to receive TAVR after detailed informed consents from the patients and their relatives.

All patients received TAVR according to standard procedure. Patients received self-expandable valves (Corevalve, Evolut R, Evlolut PRO, or Evolut PRO+; Medtronic plc., Minneapolis, Minnesota) or balloon-expandable valves (Sapien XT or Sapien 3; Edwards Lifesciences Inc., Irvine, CA, USA) via trans-femoral, trans-aorta, trans-subclavian, or direct aorta approach under general or local anesthesia support. These procedural strategies were planned by the heart-valve team conference and finally determined by the attending cardiologists.

Clinical management after TAVR was provided by the attending cardiologists. Patients were generally discharged from index hospitalization following 1 week of careful observation for procedure-related complications. After the index discharge, patients were followed at our out-patient clinic or affiliated institutions by board-certified cardiologists. Anti-platelet regimens were at the discretion of the attending cardiologist according to patient comorbidities.

### 2.4. Independent Variable and Primary Outcome

The independent variable was defined as 6MWD that was performed on index admission prior to TAVR. The primary outcome was defined as all-cause death or all-cause readmission during a 2-year observation period following index discharge.

### 2.5. Other Clinical Parameters

All clinical data used in this study were retrieved from the prospective institutional registry database. In detail, demographic, comorbidity, laboratory, and echocardiographic data obtained on admission and following TAVR were abstracted from the electrical medical chart. All-cause death and all-cause readmission dates following index discharge were also assessed and adjudicated by multiple investigators.

### 2.6. Statistical Analysis

Continuous variables were presented as median and interquartile range and compared using the Mann–Whitney U test regardless of their distribution given the moderate sample size. Categorical variables were presented as numbers and percentages and compared using Fisher’s exact test. A value of 2-tailed *p* < 0.05 was considered statistically significant. Statistical analyses were performed with SPSS Statistics 22 (SPSS Inc., Armonk, IL, USA).

The independent variable was defined as baseline 6MWD. The dependent variable (primary endpoint) was defined as a composite of all-cause death and all-cause readmission during a 2-year observation period after index discharge (day 0). The effect of baseline 6MWD on the primary endpoint was assessed using Cox proportional hazard ratio regression analyses. Variables that were significantly different between the two groups stratified by 6MWD cutoff were included in the univariable Cox analyses. Variables significant in the univariable analyses were included in the multivariable analyses using a frothed method to investigate the independent prognostic impact of 6MWD.

## 3. Results

### 3.1. Baseline Characteristics

A total of 352 patients who were registered in our institutional database were screened for inclusion in the study. Of them, 47 patients were excluded because they could not tolerate 6MWT. Patients who died during the index hospitalization and those with lost follow-up were excluded. After initial screening, a total of 299 patients were eligible for inclusion (Table 2). The median age was 86 (83, 89) years and 85 (28%) were men. The median STS score was 4.7 (3.9, 6.2). No patients had peripheral arterial disease or a history of disabling stroke. Median glomerular filtration rate was 48 (37, 60) mL/min/1.73 m^2^ and median plasma B-type natriuretic peptide level was 220 (119, 477) pg/mL.

### 3.2. Baseline 6MWD

**Figure 1:**
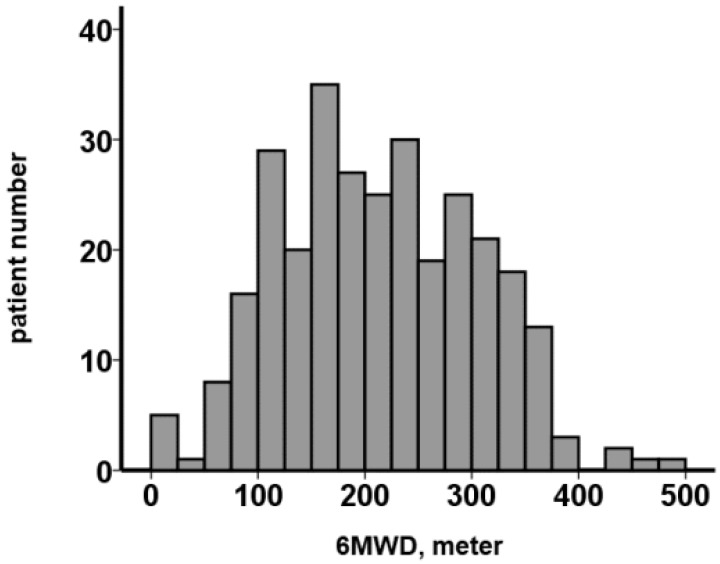
Distribution of 6MWD.

The 6MWT was performed on all participants on admission of index hospitalization. The 6MWD distributed widely, with a median value of 204 (143, 282) meters (Figure 1). Patients were divided into two groups according to the cutoff of 6MWD, which was statistically calculated as detailed below.

The 6MWD is 6-min walk distance. The 6MWD was measured at baseline before TAVR on admission of index hospitalization. The 6MWD distributed widely, with a median value of 204 m.

### 3.3. Clinical Variables Stratified by the Cutoff of 6MWD

Several baseline variables significantly differed by the cutoff of 6MWD. A shorter 6MWD was associated with older age, more prevalent with women, lower baseline hemoglobin, lower baseline glomerular filtration rate, and higher plasma B-type natriuretic peptide level (*p* < 0.05 for all; Table 2).

TAVRs were successfully performed in all participants. All patients tolerated the procedure and were able to be discharged. Median in-hospital duration was significantly longer in patients with shorter 6MWD compared with those with longer 6MWD: 18 (12, 29) days versus 14 (12, 22) days (*p* = 0.009). Major echocardiographic parameters including peri-valvular leak were not significantly different between those with shorter 6MWD and longer 6MWD (Table 3). Plasma B-type natriuretic peptide level was significantly higher in patients with shorter 6MWD (*p* = 0.001). The incidence of 30-day stroke and pacemaker implantation was not significantly different between the two groups (*p* > 0.05 for both).

### 3.4. Prognostic Impact of Shorter 6MWD

During a median of 730 (354, 730) days of the follow-up period, 21 patients died and 68 patients were hospitalized. As for the causes of death, there were six pneumonias, two renal failures, two infectious endocarditis, one sudden death, one stroke, one malignancy, and eight of unknown origin. In addition to 6MWD, variables that were significantly different between the two groups in Table 2 were included in the univariable time-to-event analyses for the primary outcome (Table 4): age, sex, body surface area, history of coronary artery disease, heart failure, hemoglobin, serum albumin, glomerular filtration rate, and plasma B-type natriuretic peptide level. The 6MWD (per 100 m) was significantly associated with the primary outcome with an unadjusted hazard ratio of 0.68 (95% confidence interval 0.52–0.88, *p* = 0.004). In the multivariable analyses, 6MWD (per 100 m) tended to be associated with the primary outcome with an adjusted hazard ratio of 0.76 (95% confidence interval 0.58–1.01, *p* = 0.055) (Table 4).

**Figure 2:**
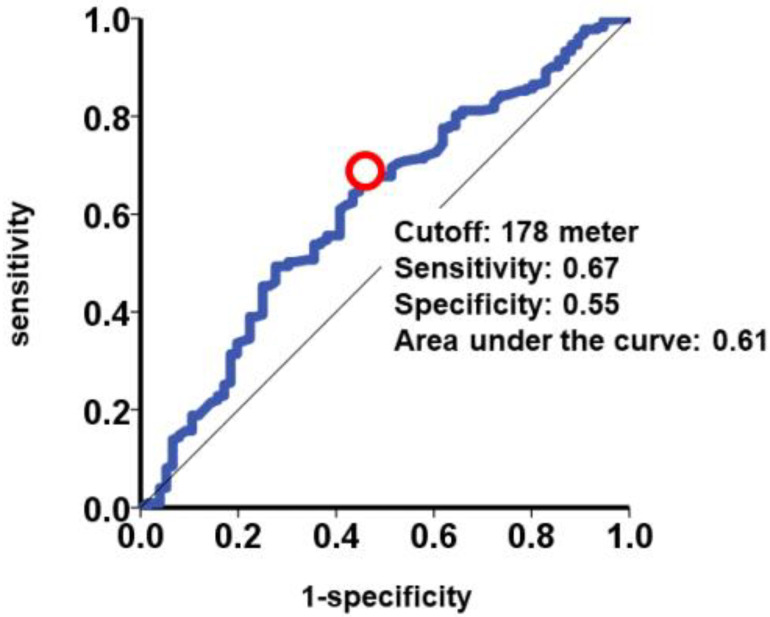
Receiver operating characteristics analysis for 6MWD to estimate primary outcome. 6MWD, 6-min walk distance. Cutoff of baseline 6MWD to best discriminate the primary outcome was calculated as 178 m at a red circle.

**Figure 3:**
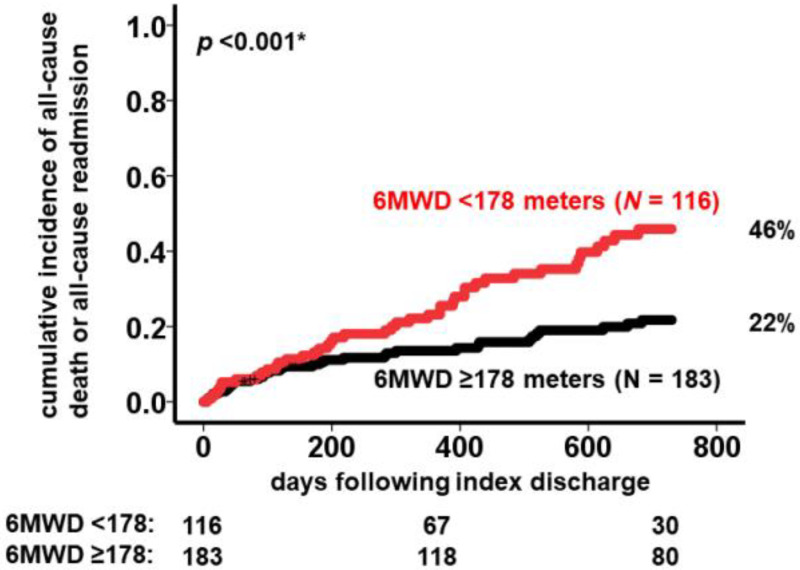
Cumulative incidence of the composite endpoint consisting of all-cause death and all-cause readmission during 2-year observational period following index discharge. 6MWD, 6-min walk distance. A cumulative incidence of composite endpoint was higher in patients with shorter 6MWD compared with those with longer 6MWD in 24%. * *p* < 0.05 by log-rank test.

In the receiver operating characteristics analysis, a cutoff for 6MWD to predict the primary outcome was calculated to be 178 m (Figure 2). Patients with a 6MWD < 178 m (N = 116) had a significantly higher cumulative incidence of the primary outcome compared to patients with a 6MWD ≥ 178 m (N = 183) (46% versus 22%, *p* < 0.001; Figure 3).

## 4. Discussion

In this analysis, we examined the effect of baseline 6MWD on the composite of all-cause death and all-cause readmission after TAVR during a 2-year observation period. A median value of 6MWD at baseline before TAVR was 204 m. Baseline 6MWD was associated with post-TAVR death or readmission during 2-year observation period after index discharge with a cutoff of 178 m of baseline 6MWD, although the association did not reach statistical significance in the multivariable analysis.

### 4.1. 6MWD and Frailty

The 6MWT is a safe and convenient method to assess functional capacity [8]. The 6MWD correlates well with peak oxygen consumption and can be easily applied to estimate prognosis in patients with a variety of comorbidities and etiologies including chronic heart failure [9]. It is a method that can be applied to better identify the presence of frailty [10], which may be common in patients with severe aortic stenosis, as the incidence often correlates with age [5]. Approximately half of the patients in this study had a 6MWD < 200 m, indicating severe baseline functional limitations [8].

### 4.2. Frailty-Related Index in TAVR Candidates

Given the complexity of the concept of frailty, there is currently no widely accepted consensus on of what constitutes frailty [11]. A variety of frailty indices have been introduced and applied to TAVR candidates, including frailty score [12]. multi-dimensional geriatric assessment [6], and an essential frailty toolset [13]. These assessments are relatively complex which may limit the ability for clinicians to easily implement prior to TAVR. Others, such as clinical frailty scale and Katz index [5,14], are simple but might be subjective. Methods not incorporating functional assessment, including geriatric nutritional risk index and muscle fat index, both of which assess malnutrition and sarcopenia, respectively, may be useful in TAVR candidates to predict clinical outcomes [15,16].

6MWD is advantageous given its ease of implementation and objective result which carries validated prognostic information [8]. As discussed in previous literature of the prognostic impact of frailty-related indexes in TAVR candidates, frailty-related comorbidities are expected to have aggregate prognostic impact and increase the incidence of all-cause mortality and morbidity following TAVR [5].

A previous study also examined the prognostic impact of 6MWD on post-TAVR outcomes [17], with no difference in 30-day outcomes or mortality when stratified by baseline 6MWD levels. This study included patients at high surgical risk with a mean STS score >10. The indication for TAVR has now been expanded to include those at lower surgical risk; we included those at low or intermediate surgical risk with a median STS score of 4.7.

### 4.3. Clinical Implications of Our Results

In light of our findings, patients with extremely short baseline 6MWD should be given special attention or the procedure should be reconsidered, given their higher mortality and morbidity after TAVR. Efforts to best address functional capacity in TAVR candidates should be prioritized, given the expected higher risk of more post-operative complications in those with poor baseline functional status [18]. We excluded those who were intolerant of 6MWT due to their comorbidities and advanced frailty. The 6MWT would have several limitations in its interpretation and applicability.

### 4.4. Limitations

This is a retrospective study consisting of a moderate sample size. Several statistics might have reached significant levels if the sample size had been further increased. We performed multivariable analyses, but other unadjusted potential confounders may also have affected the risk of the primary endpoint. Of note, 6MWD did not reach statistical significance in the multivariable analysis, probably due to the small number of the clinical events. In addition, 6MWT may be affected by various confounders that should be considered, including peripheral artery disease, pulmonary function, cognition, vision, and test protocol [8]. We excluded patients with peripheral artery disease and pulmonary disease to minimize such limitations. Therefore, special care should be taken when applying our findings using 6MWD to other cohorts, especially those with these comorbidities. We focused on 6MWD and did not examine its association with other exercise- or frailty-related parameters. We measured 6MWD once before TAVR, and its trend after TAVR remains unstudied [19]. Given the multiple causes of death, we could not assess detailed associations between 6MWD and each cause of death.

## 5. Conclusions

The 6MWT can be performed safely and easily in most TAVR candidates to assess functional capacity. Many current TAVR candidates have a relatively shorter 6MWD, indicating advanced frailty. A shorter 6MWD at baseline before TAVR was associated with mortality and morbidity following TAVR in patients with severe aortic stenosis during the mid-term observation period. The 6MWT should be a practical method to assess functional capacity and predict clinical outcomes when considering TAVR in patients with severe aortic stenosis. The clinical implications of aggressive cardiac rehabilitation to improve 6MWD and 6MWD-guided optimal patient selection, as well as the applicability of our findings to other cohorts, remain the next concern.

## Figures and Tables

**Table 1:** Inclusion and exclusion criteria.

**Inclusion criteria**
Patients who finally received successful TAVR
Age over 18 years old
Patients who agreed to participate in this study
**Exclusion criteria**
Patients unable to tolerate 6MWT due to preexisting comorbidity including stroke, PAD, and pulmonary diseases
Patients unable to tolerate 6MWT due to advanced frailty, sarcopenia, and malnutrition
Patients with significant symptom due to aortic stenosis
Patients with unstable hemodynamics
Patients receiving continuous intravenous inotropes
Patients who died during the index hospitalization
Patients who were lost follow-up
Patients with data lack

TAVR, trans-catheter aortic valve replacement; PAD, peripheral arterial disease; 6MWT, six-minute walk test.

**Table 2:** Baseline characteristics before TAVR.

	Total (N = 299)	6MWD < 178 m (N = 116)	6MWD ≥ 178 m (N = 183)	*p* Value
Demographics				
Age, years	86 (83, 89)	87 (84, 90)	85 (81, 88)	<0.001 *
Men	85 (28%)	26 (22%)	59 (32%)	0.043 *
Body surface area, m^2^	1.38 (1.29, 1.51)	1.36 (1.24, 1.46)	1.40 (1.31, 1.52)	0.006 *
Systolic blood pressure, mmHg	118 (106, 128)	117 (103, 128)	118 (107, 129)	0.47
Pulse rate, bpm	70 (63, 78)	70 (65, 77)	70 (63, 78)	0.52
STS score	4.7 (3.9, 6.2)	4.7 (3.9, 7.5)	4.6 (3.8, 5.8)	0.20
Comorbidity				
Hypertension	223 (75%)	85 (73%)	138 (75%)	0.39
Diabetes mellitus	57 (19%)	18 (16%)	39 (21%)	0.14
Dyslipidemia	149 (50%)	54 (47%)	95 (52%)	0.22
Atrial fibrillation	39 (13%)	19 (16%)	20 (11%)	0.12
Coronary heart disease	74 (25%)	37 (32%)	37 (20%)	0.017 *
History of stroke	41 (14%)	19 (16%)	22 (12%)	0.18
History of heart failure	115 (38%)	57 (49%)	58 (32%)	0.002 *
Laboratory data				
Hemoglobin, g/dL	11.4 (10.1, 12.4)	10.8 (9.9, 12.0)	11.6 (10.3, 12.5)	0.001 *
Serum albumin, g/dL	3.8 (3.5, 4.0)	3.6 (3.4, 3.9)	3.9 (3.6, 4.1)	<0.001 *
eGFR, mL/min/1.73 m^2^	48 (37, 60)	47 (35, 57)	50 (38, 64)	0.047 *
Plasma B-type natriuretic peptide, pg/mL	220 (119, 477)	353 (156, 580)	177 (101, 379)	<0.001 *
Echocardiography				
Aortic valve peak velocity, m/s	4.4 (4.0, 4.9)	4.4 (4.0, 4.9)	4.4 (4.0, 4.8)	0.87
Left ventricular end-diastolic diameter, mm	45 (41, 50)	45 (40, 50)	46 (42, 50)	0.30
Left ventricular ejection fraction, %	65 (54, 70)	63 (53, 69)	66 (55, 71)	0.083

6MWD, six-minute walk distance; STS, society of thoracic surgeons; eGFR, estimated glomerular filtration rate. Continuous variables are stated as median and interquartile and compared between the two groups using Mann–Whitney U test. Categorical variables are stated as number and percentage and compared between the two groups using Fischer’s exact test. * *p* < 0.05.

**Table 3:** Post-procedural data.

	6MWD < 178 m	6MWD ≥ 178 m	*p* Value
Plasma B-type natriuretic peptide, pg/mL	173 (90, 329)	80 (49, 173)	0.001 *
Left ventricular ejection fraction, %	64 (57, 73)	67 (61, 73)	0.65
Peak velocity at aortic valve, m/s	2.2 (1.8, 2.4)	2.1 (1.8, 2.4)	0.49
Peri-valvular leak	0	3 (2%)	0.17
Stroke within 30 days	2 (2%)	0	0.08
Pacemaker implantation within 30 days	10 (9%)	13 (7%)	0.39

6MWD, six-minute walk distance. Continuous variables are stated as median and interquartile and compared between the two groups using Mann–Whitney U test. Categorical variables are stated as number and percentage and compared between the two groups using Fischer’s exact test. * *p* < 0.05.

**Table 4:** Prognostic impact of clinical variables on the primary endpoint.

	Univariable Analyses		Multivariable Analyses	
	Hazard Ratio (95% CI)	*p* Value	Hazard Ratio (95% CI)	*p* Value
Age, years	1.01 (0.97–1.06)	0.61		
Men	0.65 (0.41–1.04)	0.075		
Body surface area, m^2^	0.97 (0.25–3.80)	0.97		
Coronary artery disease	1.23 (0.74–2.03)	0.42		
History of heart failure	1.95 (0.96–3.07)	0.068		
Hemoglobin, g/dL	0.82 (0.71–0.94)	0.006 *	0.90 (0.77–1.05)	0.17
Serum albumin, g/dL	0.43 (0.26–1.04)	0.058		
eGFR, mL/min/1.73 m^2^	0.98 (0.97–0.99)	0.007 *	0.99 (0.98–1.01)	0.15
Plasma B-type natriuretic peptide, ×100 pg/mL	1.82 (1.11–3.00)	0.018 *	1.40 (0.83–2.37)	0.21
6MWD, ×100 m	0.68 (0.52–0.88)	0.004 *	0.76 (0.58–1.01)	0.055

CI, confidence interval; eGFR, estimated glomerular filtration rate; 6MWD, six-minute walk distance. Variables that were significantly different between the two groups in Table 1, in addition to 6MWED, were included in the univariable analyses. Variables that were significant in univariable analyses were included in the multivariable analysis with forced method. * *p* < 0.05.

## Data Availability

Data are available from the corresponding author upon reasonable requests.
